# Kv1.3 Ion Channels Mediate Electrical Stimulation-Induced Collagen Expression in Human Dermal Fibroblasts

**DOI:** 10.3390/cosmetics12030086

**Published:** 2025-04-23

**Authors:** Catherine Obiajulu, Diem Nguyen, Kim Hoang Ngan Bui, Timothy Tran, Annamarie Vu, Cortney Ngo, Ian A. Slowinski, Kazuyuki Miyazawa, Katarzyna Slowinska

**Affiliations:** 1 Department of Chemistry and Biochemistry, California State University Long Beach, 1250 Bellflower Blvd, Long Beach, CA 90840, USA; 2 Department of Mechanical Engineering, Purdue University, 585 Purdue Mall, West Lafayette, IN 47907, USA; 3 Mirai Technology Institute, Shiseido Co., Ltd., 1-2-11, Takashima, Nishi-ku, Yokohama 220-0011, Kanagawa, Japan

**Keywords:** dermal fibroblast, electrical stimulation, ion channels, Kv1.3, calcium imaging, collagen

## Abstract

Electrical stimulation of the skin has proven effective in pain management and antibacterial treatment, particularly in wound healing and counteracting the aging processes. The latter processes rely on epidermal cell migration, increased fibroblast proliferation, and upregulation of extracellular matrix protein expression. While an electrical field stimulates these processes, it is unclear how the electrical signal results in transcriptional control. Here, we postulate that the activation of voltage-gated channels, specifically voltage-gated potassium channels Kv1.3, is implicated in initiating the downstream signaling pathways that lead to increased collagen expression. We postulate that Kv1.3 and possibly calcium-activated potassium channel activity leads to the engagement of store-operated calcium channels and modulates the intracellular calcium ions distribution. In turn, changes in intracellular calcium concentration can activate calcium-generated transcriptional effectors. The Kv1.3 channel, identified via fluorescence imaging with ShK toxin (peptide), shows high-level expression in the human dermal fibroblast cell membrane. We also performed proliferation, collagen expression, and calcium imaging studies for variable electrical fields to help understand the link between the electrical stimulation, Kv1.3 channels, intracellular calcium concentration, and protein expression.

## 1. Introduction

Electrical stimulation (ES) of the skin has emerged as a promising therapeutic approach in medical and cosmetic applications [1–3]. It has been shown to effectively manage pain, provide antibacterial treatment, and significantly enhance wound healing [4–7]. Furthermore, its observed effects in counteracting the natural aging processes of the skin, ease of application, and safety have resulted in broad interest in using ES in cosmetic science [8–10].

ES, specifically non-invasive types like Electrical Muscle Stimulation, microcurrent, and radiofrequency, is often used in cosmetic treatments for its non-therapeutic, aesthetic benefits. These cosmetic effects do not treat medical conditions but aim to enhance appearance. ES has been shown to improve muscle toning and lifting, skin tightness and circulation, reduce the appearance of fine lines, and help with better product adsorption [3,10–13].

The beneficial effects of ES are primarily attributed to its ability to influence critical biological mechanisms. Specifically, electrical stimulation promotes epidermal cell migration, improves muscle toning, stimulates the proliferation of fibroblasts (affects the cell cycle), and enhances the expression of extracellular matrix proteins [4,14]. These cellular and molecular responses play a vital role in skin regeneration, tissue repair, and maintaining the skin’s structural integrity [15].

While the link between the ES and increased extracellular matrix expression in Human Dermal Fibroblasts has been established, the transduction mechanism resulting in increased transcription after ES is unclear [16]. The lipid bilayer of the cell membrane acts as a dielectric between two conductive environments (the intracellular and extracellular fluids) with frequency-dependent impedance in the range between 1 and 100 MΩ for low frequencies (below 1 kHz) [17,18]. The high impedance of the cell membrane protects the cell from environmental factors and prevents the penetration of the electric field. Thus, we postulate that the first step is electrical signal transduction and activation of the voltage-gated ion channels due to membrane polarization during the exposure to the electric field. Subsequently, activation of voltage-gated ion channels results in modulation of intracellular calcium ion distribution; calcium ions are known secondary messengers that actively participate in transcriptional control [19,20]. Thus, the final effect of ES is the increased expression of extracellular matrix proteins [16].

Human Dermal Fibroblast cells belong to the family of non-excitable cells [21–24]. The most prevalent voltage-gated ion channels present in Human Dermal Fibroblast cell membranes are voltage-gated potassium channels (Kv) [25,26]. Kv channels are a large family of both slow- and fast-acting channels present in both excitable and non-excitable cells. It has been shown that the upregulation of Kv1.3 channels is specifically linked to increased fibrosis and cell proliferation in cardiac fibroblast cells [26]. Kv1.3 channels are also implicated in the increased proliferation rate of malignant cells [27,28]. Due to the recently established link between the Kv1.3 channels and increased expression of proteins and proliferation in several different cell types related to Human Dermal Fibroblasts, we postulate that Kv1.3 is the channel present in Human Dermal Fibroblasts and responsible for signaling the presence of electric field.

Teisseyre et al. [27] propose two distinct mechanisms where Kv1.3 channel activation can increase cell proliferation and protein expression. The distinctive difference between the models is the required presence of the calcium-activated potassium channels (specifically, large-conductance calcium-activated potassium channels) in the “membrane potential model”. The calcium-activated potassium channels are voltage-gated and are the most commonly expressed channels in Human Dermal Fibroblasts [25]; thus, we postulate that Kv1.3 and calcium-activated potassium channels participate in Human Dermal Fibroblasts activation during the electrical stimulation via a mechanism similar to the “membrane potential model”. In that model, one of the forward steps in activation is calcium influx as a cellular response to potassium outflux.

The calcium influx via store-operated calcium channels is a common mechanism of intramolecular free calcium concentration modulation in cells [20,29]. The changes in intracellular calcium concentration control many regulatory processes since calcium is a secondary messenger. One of the processes that is controlled by calcium ions is transcriptional control. Over the past decade, research has identified three main mechanisms by which Ca^2+^ sensors respond to changes in intracellular free Ca^2+^ levels: (1) activation of Ca^2+^-dependent kinases and phosphatases, which alter transcription factors’ activity, nuclear localization, or cofactor interactions, affecting nucleosomal properties and gene accessibility; (2) direct changes in calcium sensor binding to specific DNA sites due to Ca^2+^ binding, influencing transcriptional processes; and (3) Ca^2+^-dependent protein–protein interactions between calcium sensors and transcription factors or other proteins in the enhanceosome, modifying DNA binding or cofactor recruitment to regulate transcription [19].

Regardless of the transcription regulatory pathway, the calcium exchange across the cell membrane is a crucial step in all mechanisms [30,31]. Recently, a detailed study by Schleinhege et al. [32] showed that the presence of ORAI1, a protein located on the plasma membrane that acts as a store-operated calcium channel, leads to increased deposition of collagen in pancreatic stellate cells that produce a collagen-rich connective tissue in chronic pancreatitis and pancreatic ductal adenocarcinoma.

The purpose of this study is to elucidate the molecular mechanisms by which electrical stimulation influences collagen expression, with a particular focus on the signal transduction pathways that mediate the conversion of external electric fields into intracellular signals. These pathways ultimately lead to the activation of specific transcription factors involved in the regulation of collagen gene expression. Here, we postulate that the first step in Human Dermal Fibroblast activation leading to an increase in the expression of collagen by an electric field is the opening of the Kv1.3 channel that leads to the changes in intracellular calcium concentration that is implicated in the transcriptional control.

## 2. Materials and Methods

### 2.1. Materials and Reagents

All reagents and materials were used as purchased (in the purest form available) unless stated otherwise. ShK and 5FAM-ShK (Bachem AG, Bubendorf, Switzerland), Fluo-4 (Invitrogen, Carlsbad, CA, USA), 4-aminopyridine [4AP] (Sigma, St. Louis, MO, USA), Psoralen [Pap-1] (Sigma, St. Louis, MO, USA).

### 2.2. Cell Culture

The Human Dermal Fibroblast cells were purchased from the American Type Culture Collection (ATCC, Manassas, VA, USA) and cultured in a T-25 flask according to the specifications from ATCC. The Human Dermal Fibroblast cells were cultured in Fibroblast Basal Medium (ATCC) supplemented with Fibroblast Growth Kit- Low Serum (ATCC) and 0.5% Penicillin Streptomycin L-Glutamine Mixture (Pen/Strep, Lonza, Morristown, NJ, USA). Once the cells reached an 80% or higher percentage confluence, they were counted and seeded (5.0 × 10^4^ cells/cm^2^) on a 35 mm Petri dish (Mattek, Ashland, MA, USA) or seeded on 1 cm^2^ polylysine slides (Thermo Fisher Scientific, Waltham, MA, USA) in 60 mm Petri dishes (Corning, Glendale, AZ, USA). Passages 3–10 were used for all experiments.

### 2.3. Electrical Stimulation of Cells

A reactor for electrical stimulation was assembled, as described by Tandon et al. [33], using a petri dish (100 mm), two carbon rods, and platinum wires. The platinum wires were attached to the carbon rods and glued 1 cm apart into a petri dish. Three polylysine slides with cells (90% or higher confluence) were transferred into the reactor. The reactor was transferred to a 37 °C incubator and attached to Tektronix AFG 31000 Arbitrary Function Generator. For microscopy, the reactor design was changed: it consisted of a 35 mm Petri dish with a glass cover slip (Mattek), where the lid was modified with two glassy carbon electrodes extending to the bottom of the dish, either made in-house or purchased custom made from IonOptix (Westwood, MA, USA). The cells were stimulated with the pulsed electric field where the applied amplitude voltage was from 0 to up to +10 V, using pulse mode 1–2 kHz with a 10% duty cycle.

### 2.4. Quantification of Collagen Concentration

The Human Dermal Fibroblast cell cultures on polylysine slides (Thermo Fisher Scientific, Waltham, MA, USA) were transferred from the reactor into a 35 mm petri dish (Corning), trypsinized (Gibco at Thermo Fisher Scientific, Carlsbad, CA, USA) and after neutralization of trypsin, a cell scraper was used to ensure that all cells were removed from the slides and analyzed for survival with Trypan Blue (Corning) exclusion assay and Luna II (Logos Biosystems, Annandale, VA, USA) cell counter. The cells were placed in a microcentrifuge vial and then rinsed in 1 mL PBS three times. To determine the intracellular concentration of collagen, Human Dermal Fibroblast cells were lysed using 0.5 mL RIPA (Thermo Scientific) buffer for 1 h followed by centrifugation at 13,000 rpm for 10 min.

The Sircol soluble collagen assay kit was employed, and the protocol provided by Biocolor Ltd. (Belfast, UK) was followed to determine the intracellular concentration of procollagen. The Sircol assay is designed to detect acid-soluble and pepsin-soluble collagens, including newly synthesized collagens. It is a dye-binding method where the Sircol Dye reagent binds to the [Gly-X-Y]_n_ helical structure as found in collagens. The method is most specific to mammalian collagens type I-V.

1.0 mL of Sircol Dye Reagent was added to each tube with 100 μL of the sample. The tubes were capped and manually mixed at 5-min intervals for 30 min. The tubes were then transferred to a microcentrifuge, and unbound dye was removed by aspiration. A volume of 750 μL ice-cold Acid–Salt Wash Reagent was added, and the tubes were transferred to a microcentrifuge to spin at 12,000 rpm for 10 min. The acid–salt was removed by aspiration, and 250 μL alkali reagent was added to each tube. The collagen-bound dye was released into the solution using a vortex mixture. A volume of 200 μL from each tube was then transferred to a 96-well plate and read at 555 nm using a plate reader (Thermo Electron Corporation, Waltham, MA, USA). The quantification was determined in triplicates and compared to a control in which the cells were not electrically stimulated. The concentration of collagen produced was determined by comparison to a standard curve and normalized by the number of cells that were lysed for the assay.

### 2.5. Microscopy

The morphology and confluency of the Human Dermal Fibroblasts were studied using an inverted light/fluorescence microscope (Nikon Eclipse Ti2, Nikon Instruments Inc. Melville, NY, USA), and time-lapse fluorescence images were collected with EVOS M5000 Cell Imager (Thermo Fisher Scientific, Waltham, MA, USA). The fluorescence images were collected on live cells stained with the desired fluorescence dye (5FAM-ShK, Fluo-4), following the protocols provided by the vendor (Fluo-4) or following the protocol for flow cytometry (5-FAM-ShK), see below. The cells were imaged in Live Cell Imaging Solution (Invitrogen). The time-lapse images were collected as fast as the image acquisition software allowed, and the actual time differences between frames were calculated using metadata. The image analysis was performed with software (Python, v.2024.12.1) written by I.A. Slowinski by selecting the Region-Of-Interest and average fluorescence intensity per pixel in time evolution in each frame. The used software is available upon request.

### 2.6. Proliferation Assay

After reaching 70–80% confluency, Human Dermal Fibroblast cells were detached from the cell culture flask with Accutase (Gibco) and counted (Luna II cell counter, Logos Biosystems). Cells were seeded into a 96-well plate at 5 × 10^3^ cells/well and incubated for 1 h. The proliferation assay was administered after 1 h and 24 h. In short, 100 μL of CellTiter-Glo 2.0 (Promega, Madison, WI, USA) reagent was added to each well, and the plate was inserted into a plate reader (BioTek Synergy H1, Agilent, Santa Clara, CA, USA) and mildly shaken for 2 min. The readout was set for a 10-min delay and read in luminescence mode.

### 2.7. Flow Cytometry

Human Dermal Fibroblast cells were incubated in complete media with 5FAM-ShK peptide (9.864 × 10^−10^ M stock solution in Ultra-Pure Distilled Water, MilliQ, Millipore Sigma, Burlington, MA, USA) for different time intervals from 5 to 60 min in a set of final concentrations between 5 and 50 pM. After incubation with 5FAM-ShK peptide, cells were dislodged by trypsin, washed three times with cold PBS (spun at 1000 rpm for 2 min), and then resuspended in PBS containing 0.1% BSA. Cells (50,000/sample) were analyzed using flow cytometry (Cell Lab Quanta SC-MPL, Beckman Coulter, Inc., Brea, CA, USA) to quantify the cellular presence of the peptides in the FITC channel (5-FAM).

### 2.8. Data Analysis

All measurements were performed either three or six times. The error bars in all figures represent standard deviation. Statistical significance was established based on the one-way ANOVA analysis (*p* < 0.05).

## 3. Results

### 3.1. ES Measurements

**Figure 1.**
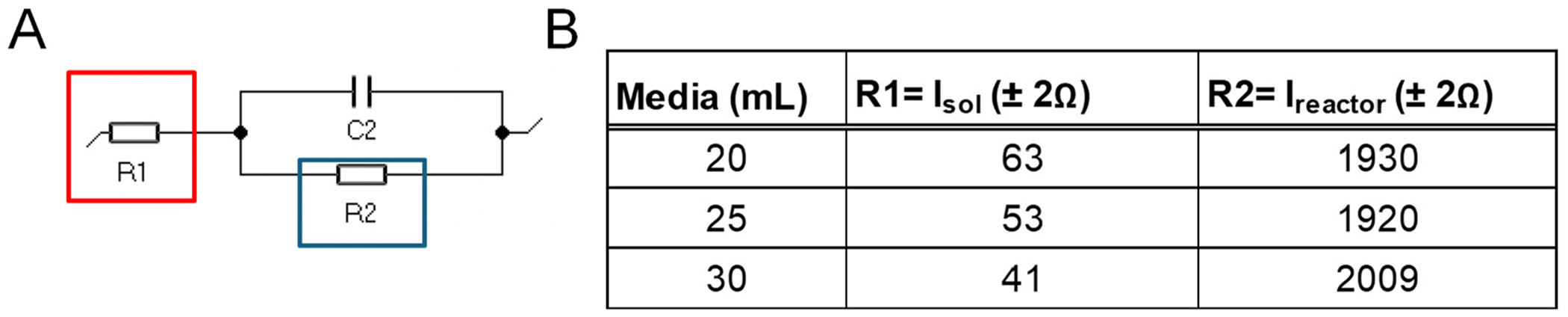
Equivalent circuit diagram for an ES reactor (**A**). The impedance of the circuit elements; R2 represents the reactor impedance (**B**).

ES is a broad term for using either the voltage-controlled or current-controlled electrical signal to measure the changes in cells affected by the stimulation. Here, we measured the changes in concentration of collagen that represent Extra Cellular Matrix proteins before and after administrating the voltage pulse in the two-electrode system. The details of the measurement conditions have been published in our previous investigation [34]. In short, in this report, we applied the pulse using positive bias (from 0 V to maximum +10 V) with 1 Hz or 1 kHz pulse mode with a 10% duty cycle. The distance between the electrodes is 1.0 cm; hence, the applied voltage is also the strength of the field in V/cm. The shape and accuracy of the delivered pulse were monitored with InstaView (real-time waveform measurement) integrated into the AFG31000 Series Arbitrary Function Generator. The reported frequency is measured from the initial time of each pulse. We constructed the ES reactor as described previously [33,34] and measured the reactor’s impedance (~2 kΩ) with the media present (Figure 1 and Figure S1). This value was used to choose the settings in the voltage application with AFG31000.

### 3.2. Collagen Expression

Human dermal fibroblasts were seeded on 1 cm^2^ polylysine-coated glass slides and, upon reaching 80–90% confluence, were placed in a bioreactor for electrical stimulation. Voltage pulses (0–10 V), frequencies (1–1 kHz), and rest times before collagen concentration was measured. Since collagen is insoluble in media, it was detected as intracellular procollagen prior to secretion. Collagen concentration was measured using the Sircol assay, which quantifies soluble procollagen through a dye-binding method. The assay was conducted on cell lysates to determine intracellular procollagen levels and was calibrated (Figure S2) using a standard collagen solution supplied by the manufacturer to ensure accuracy and consistency. After ES, fibroblasts were counted using a Luna II (automatic cell counter) to determine the total number of cells present before lysis. This step ensured that the intracellular procollagen concentrations were normalized to the cell count, providing reliable and comparable measurements of collagen content on a per-cell basis.

**Figure 2.**
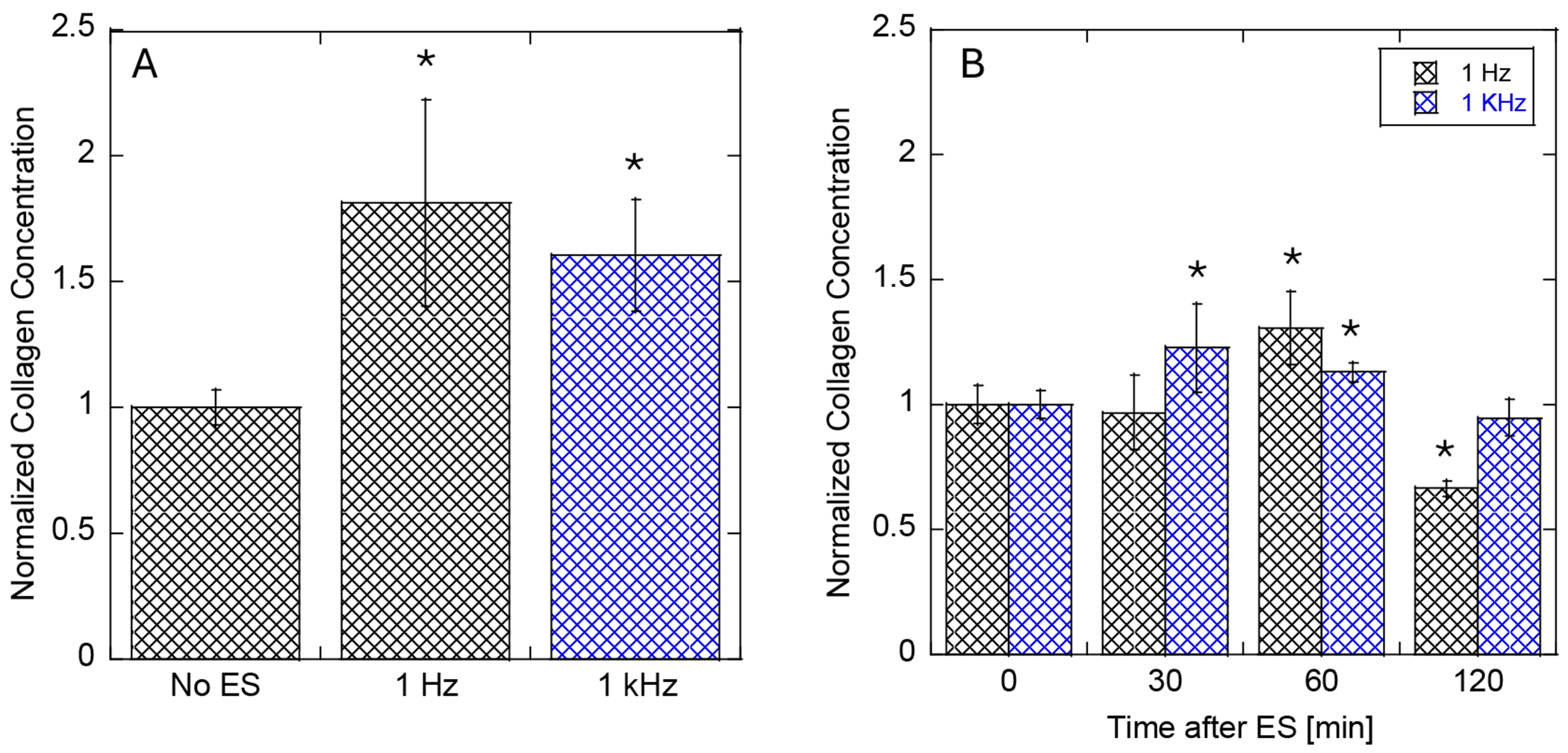
Normalized intracellular procollagen concentration in Human Dermal Fibroblasts with varying experimental conditions: (**A**) frequency dependence, with 120 min stimulation and no rest time; (**B**) rest time dependence, after 10 min stimulation for 1 Hz and 1 kHz. The applied pulse was positive bias, 5 V, 10% duty. Error bars represent the standard deviation from six measurements. * *p* < 0.05.

We have previously reported how the changes in ES conditions like applied voltage, bias, time of stimulation, and pulse time affect the collagen expression for 1 Hz pulse mode [35]. The goal of this work is twofold: (1) focus on ES conditions that are relevant in cosmetic applications: short times of ES and the possibility of the application of higher frequency (1 kHz) and (2) elucidating the possible mechanism of ES. First, we examined if the application of high frequency (1 kHz) has a similar effect to previously tested 1 Hz frequency (Figure 2A). The stimulation was performed with the 5 V positive bias for 120 min in the pulse mode with the 10% duty, and cells were immediately lysed after the ES without rest time. The procollagen expression in both 1 Hz and 1 kHz ES is increased by about 75% with no significant difference between frequencies.

In addition to long stimulation times (120 min), we have also tested the very short, 10 min ES applied to Human Dermal Fibroblasts. The procollagen synthesis kinetics are not fast enough to detect it after 10 min due to the size of the protein (about 1100 residues per chain). Thus, we applied a rest time between the end of stimulation and cell lysis. Human Dermal Fibroblast cells were lysed 30, 60, or 120 min after the end of the ES, and then the Sircol assay was performed to quantify the procollagen expression (Figure 2B). The most procollagen expression was detected after 60 min, but the levels were only about 20 to 25% higher than in non-stimulated cells for both 1 Hz and 1 kHz frequencies. After 120 min, cells return to homeostasis, and the concentration of collagen should return to the pre-stimulation level [34], and we observe this effect (Figure 2B). In addition to the Sircol assay, the change in the expression of collagen can be detected by Western blot (protein quantification) or Rt-qPCR (mRNA quantification). Both methods are very selective in the detection of specific proteins and, thus, are often used in protein identification, but the sensitivity and dynamic range of detection are not as good as the Sircol assay.

### 3.3. Identification of Kv1.3 Channel on Human Dermal Fibroblast Cell Surface

**Figure 3.**
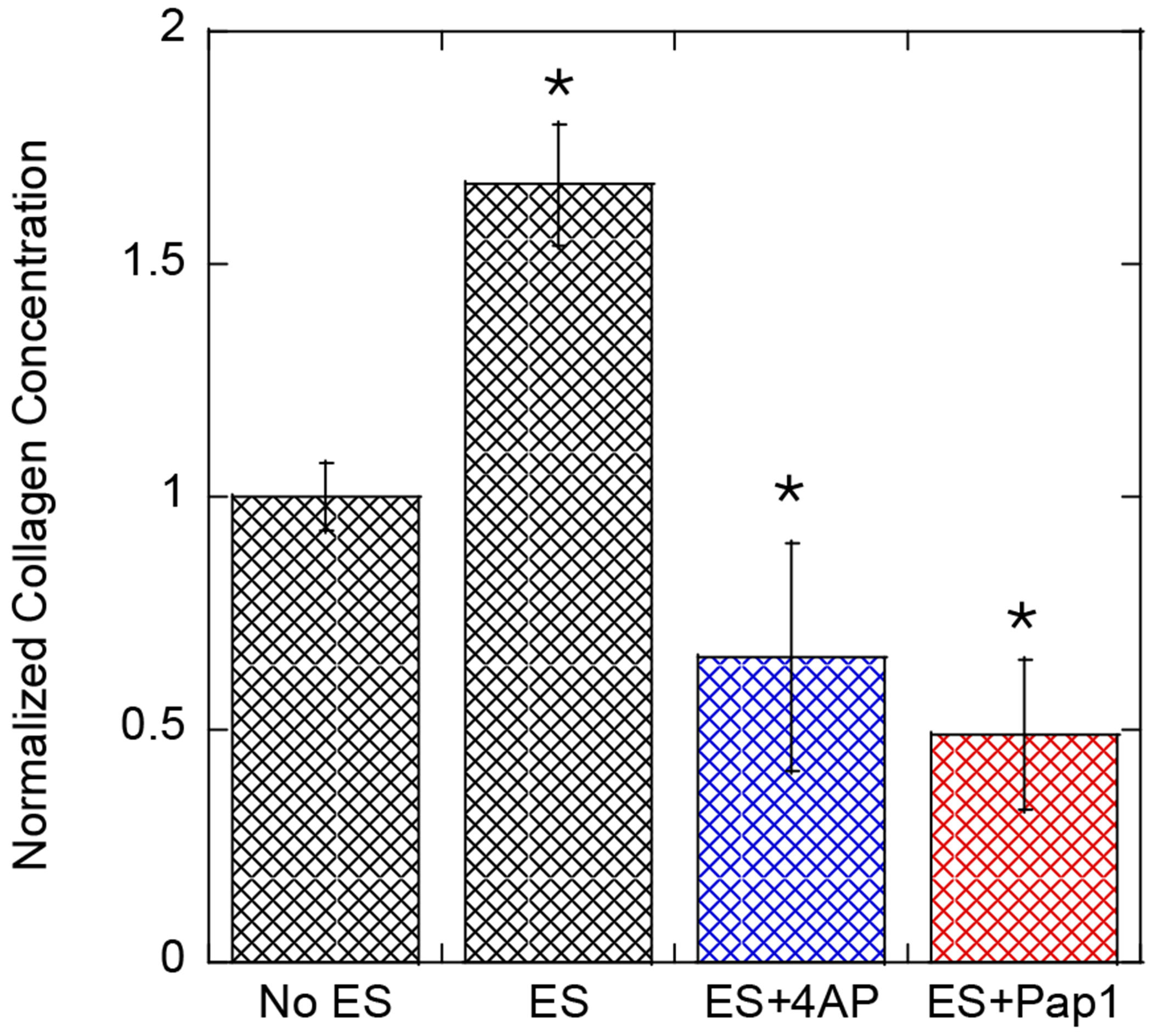
Normalized intracellular procollagen concentration in Human Dermal Fibroblasts without (no ES) and with (ES) electrical stimulation; with the addition of 20 μM of 4-aminopyridine (4AP, blue) or 5 nM of Psoralen (Pap-1, red). Error bars represent the standard deviation from six measurements. * *p* < 0.05.

The main goal of this work is to elucidate the possible mechanism of ES in Human Dermal Fibroblasts that results in upregulation of collagen expression. The ES stimulation of Human Dermal Fibroblasts is effective in increasing collagen expression. Thus, the ES signal must be transmitted to the cell effectors that regulate the transcription. The main voltage-gated ion channels found to be present on the Human Dermal Fibroblast cell surface in large quantities are the voltage-gated potassium channel (Kv) and large-conductance calcium-activated potassium channel [25]. The latter is only activated when cells encounter large membrane polarization. First, we focused on confirming that the potassium channel is indeed involved in signal transduction, which involves ES as a first step [35]. We used 4-aminopyridine, a potassium channel blocker specific for potassium channels but not specific for the type [36]. In Figure 3 (blue), we show the effect of 4-aminopyridine (20 μM) on collagen expression with ES stimulation: 20 min ES, 40 min rest time, 5 V, positive bias, 1 Hz frequency. The addition of 4-aminopyridine to the media results in a significantly lower concentration of procollagen after the ES stimulation of Human Dermal Fibroblast cells. The selectivity of 4-aminopyridine toward potassium channels is not very high (IC_50_ = 20 μM). Thus, the addition of 20 μM concentration to cells could have some off-target interactions; therefore, we intended to pick a Kv blocker that is more specific to Kv channels, avoiding off-target effects. The two most common Kv channels in fibroblast cells (all types) are Kv1 and Kv7 type channels, but Kv7 are not responsive to 4-aminopyridine; thus, we assumed that the Kv1 family is the best candidate for further investigation. We employed the small molecule, Psoralen (Psoralen), with IC_50_ = 3.45 nM towards the Kv1.3 channel to achieve better selectivity [37]. In Figure 3 (red), we show the effect of adding the 5 nM Psoralen to the cell media and electrically stimulating the cells, applying the same conditions as for the 4-aminopyridine blocker. The results are similar: the concentration of procollagen expression decreases. Because of the low concentration of Psoralen used with the comparison to 4-aminopyridine (both at the IC_50_ level for Kv1 channels), we believe that the observed effect is due to blocking the Kv1 channel, not due to off-target effects.

**Figure 4.**
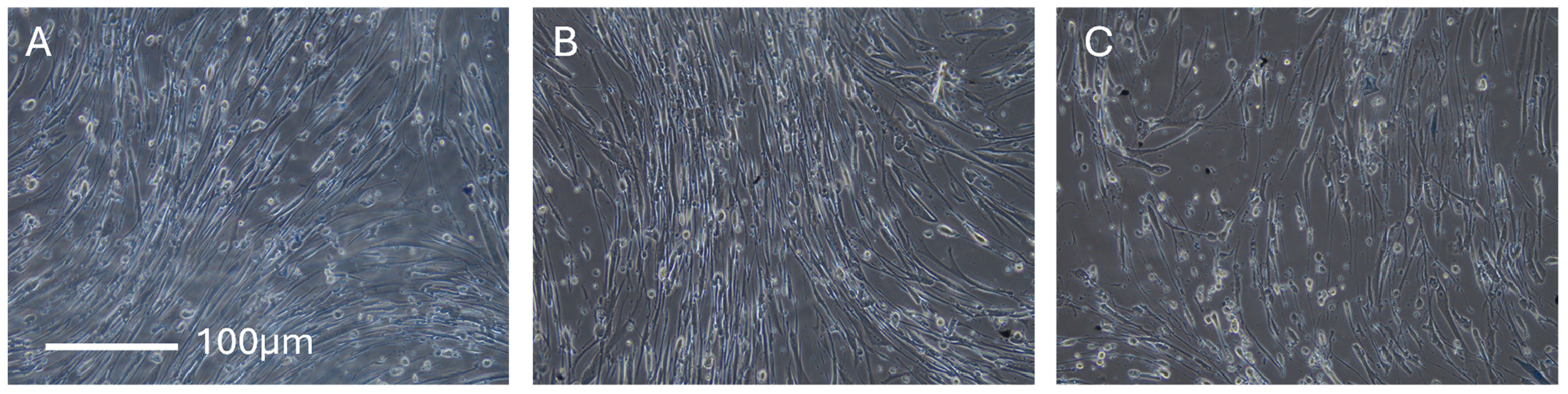
Bright-field images of Human Dermal Fibroblasts 24 h after ES (20 min, positive bias +5 V, 1% duty, 1 Hz) incubated with trypan blue. (**A**) no ES, (**B**) ES, (**C**) ES + 20 μM of 4-aminopyridine.

In addition to quantifying the collagen expression in Human Dermal Fibroblast cells treated with ES and 4-aminopyridine, we examined the cell morphology (bright field) treated with 20 μM 4-aminopyridine and ES (the same conditions). Figure 4 shows the changes in Human Dermal Fibroblast cell morphology without (Figure 4A) and with (Figure 4B,C) ES applied for 20 min (5 V positive bias, 1 Hz, 10% duty) with the image taken after 40 min of rest time. The difference between Figure 4B,C is the presence of 20 μM 4-aminopyridine in the cell media in Figure 4C. While we did not observe differences in cell morphology before and after ES (see also Figure S3), the cells treated with 4-aminopyridine and ES show shorter lamellipodia, look swollen and less elongated. The addition of trypan blue before imaging indicates that the cell membrane is intact and cells are not dead, but their morphology is not consistent with a healthy Human Dermal Fibroblast culture.

**Figure 5.**
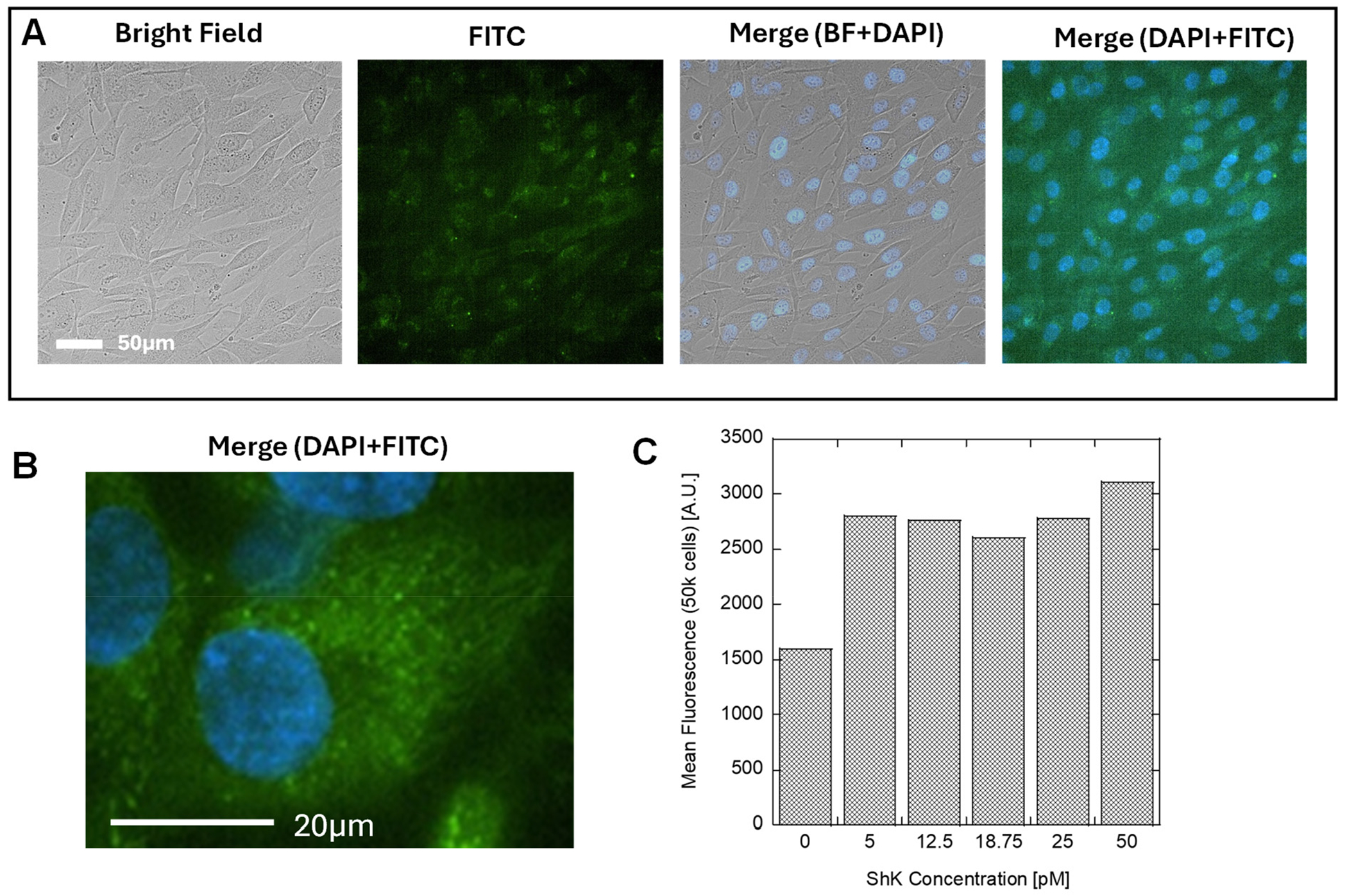
(**A,B**) Fluorescence microscopy of Human Dermal Fibroblasts incubated with 5FAM-ShK peptide (50 μM for 30 min); (**C**) Flow cytometry in FITC channel, incubated for 30 min in varying concentrations of peptide (0–50 pM, 50,000 cells/count).

To confirm that the channel that is most likely to be expressed on the cell membrane of Human Dermal Fibroblasts is the Kv1.3 channel, we applied ShK toxin, a 35-residue basic peptide from the sea anemone *Stichodactyla helianthus* that blocks specifically potassium channels with the highest affinity towards Kv1.3 (K_d_ = 11 pM) [38,39]. The ShK peptide was modified at the N-terminus with 5-carboxy fluorescein (5FAM), which enabled fluorescence microscopy imaging. We incubated Human Dermal Fibroblasts with 5FAM-ShK peptide (50 pM) for 30 min prior to imaging with a fluorescence microscope (Figure 5A,B) and analyzed the cell population (50,000) with flow cytometry (Figure 5C) depending on the concentration of ShK peptide in the media during incubation. The flow cytometry indicates the presence of Kv1.3 channels in Human Dermal Fibroblasts. The experiments with the ShK peptide, together with results obtained from experiments with 4-aminopyridine and Psoralen potassium channel blockers, are convincing proof that Kv1.3 is participating in the initial step of signal transduction between the external ES and intracellular signaling pathway.

### 3.4. Cell Proliferation

**Figure 6.**
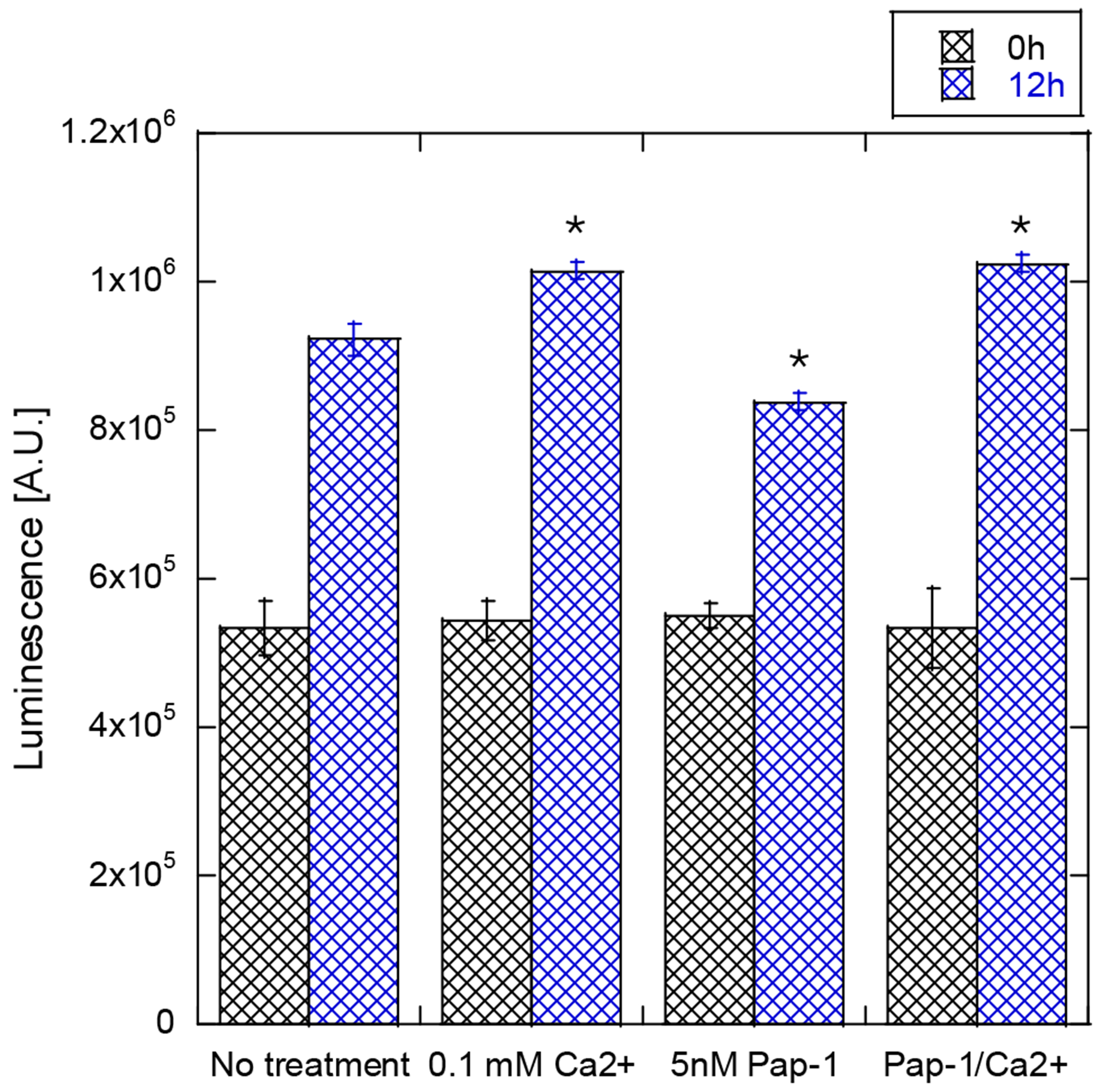
Human Dermal Fibroblasts proliferation study with Cell Titter Glo 2.0 luminescence assay. Cell counts at 0 h (black) and 12 h (blue) with different treatments of calcium and/or Psoralen (Pap-1). Error bars represent the standard deviation calculated from six measurements. * *p* < 0.05.

Recently, it has been shown that Kv1.3 channels are linked to the increase in cell proliferation in several cell types where Kv1.3 was upregulated [40]. We measured Human Dermal Fibroblast cell proliferation with partially blocked (5 nM Psoralen) Kv1.3 ion channels. For positive control, we used the elevated calcium concentration (~15%, addition of 0.1 mM) above the concentration in growth media since a moderate increase in calcium concentration in extracellular solution (or media) has been shown to stimulate cell proliferation. Figure 6 shows that the addition of Psoralen results in inhibition of Human Dermal Fibroblast cell proliferation, and the addition of calcium to the media results in an increase in cell proliferation. The treatment of Human Dermal Fibroblasts with Psoralen, along with the addition of calcium, rescues the cells and allows them to proliferate at the same rate as with the presence of calcium and absence of Psoralen.

### 3.5. Calcium Imaging

**Figure 7.**
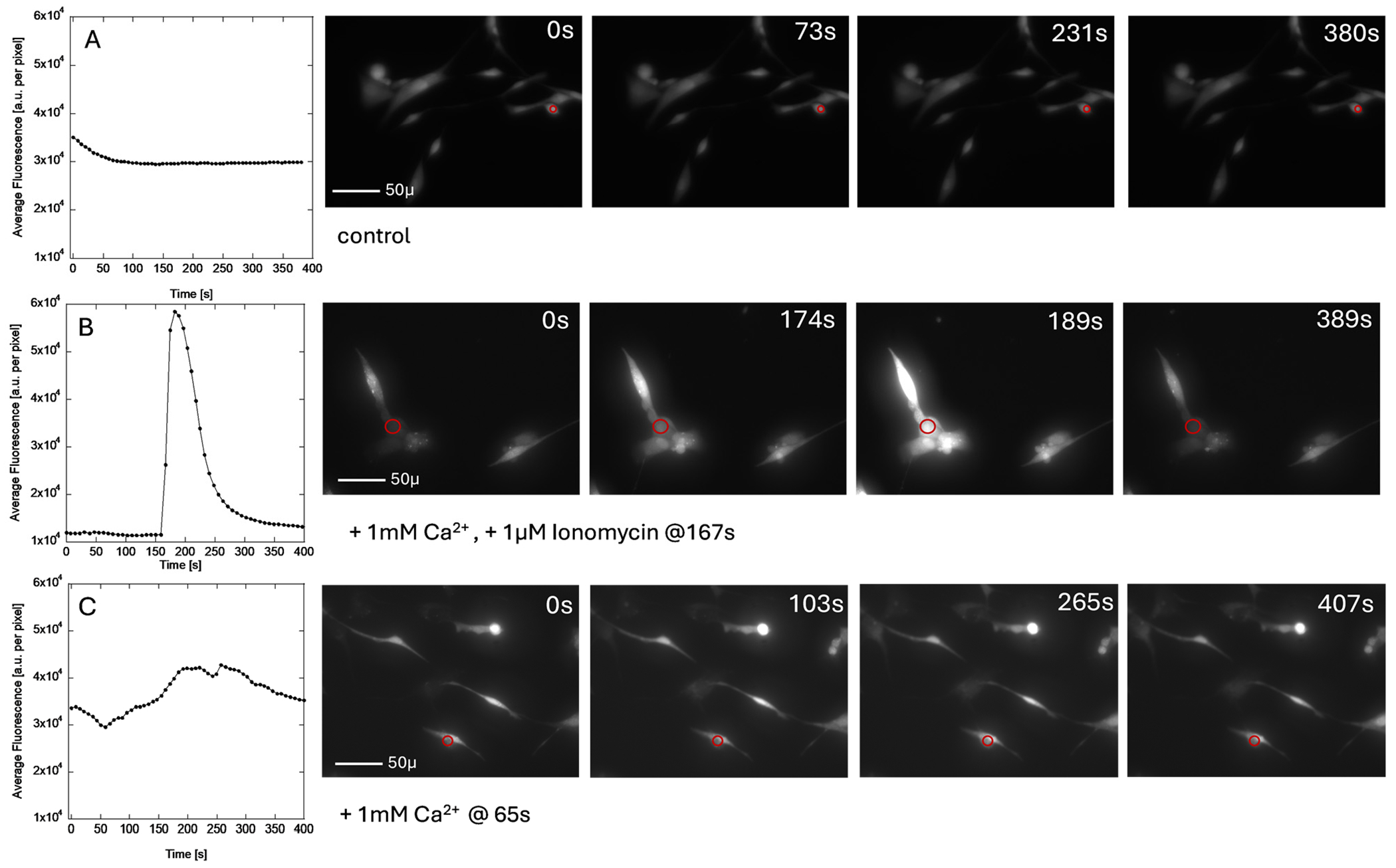
The time courses of fluorescence emission intensity in Human Dermal Fibroblast cells loaded passively with Fluo-4 averaged over the selected area (red circle) in the image plotted next to the selected time frames; (**A**) baseline, (**B**) addition of 1 μM ionomycin at 167 s, (**C**) addition of 1 mM calcium at 65 s. The images and time courses were NOT corrected for background, initial point fluorescence emission, and artificial offsets induced by the solutions added to the culture during recording. The AVI files are available as Supplementary Materials (Video S1 A–C).

Intracellular free calcium ion imaging was performed with Fluo-4 fluorescence dye [41,42]. The cells were incubated with Fluo-4 for 1 h before imaging. The images were collected on EVOS M5000 Cell Imager in a live cell imaging solution (Invitrogen) for no longer than 1 h and discarded afterward. The frames were collected (40×) as fast as EVOS software allowed (between 5 and 8 s). The actual time varied between the frames and was calculated from metadata. The measurements were calibrated with a set of control conditions (Figure 7). The negative control was no stimulation (Figure 7A). The positive controls were the addition of ionomycin (Figure 7B) and the addition of calcium ions (Figure 7C) [42]. The images shown were not digitally processed in any way or normalized. The plotted Average Fluorescence was calculated from the area indicated in the figure (red circle) using Python Script (available per request) for each frame of the time-lapse images. Each plot has the same scale for ease of comparison. The Fluo-4 fluorescence dye has a very low bleaching rate, which was observed only initially (Figure 7A). We used ionomycin (Figure 7B) to test how fluorescence emission level changes with the influx of calcium and how rapidly the intracellular concentration of calcium can be detected with the Fluo-4 dye. We were not able to plot the time evolution of the area with the highest intensity due to detector overload. Our results confirm the expected outcomes. In addition to ionomycin that disrupts the calcium cellular homeostasis, we also tested the effect of calcium addition (1 mM) to the imaging media (1.65 mM) to increase the chemical potential of calcium ions and disrupt the membrane potential due to calcium gradient across the membrane (Figure 7C). The addition of calcium to the imaging solution at 65 s results in an increase in calcium concentration in the cell that is less rapid than in the case of ionomycin, but it decreases in a similar time scale as in the case of ionomycin to the initial levels.

**Figure 8.**
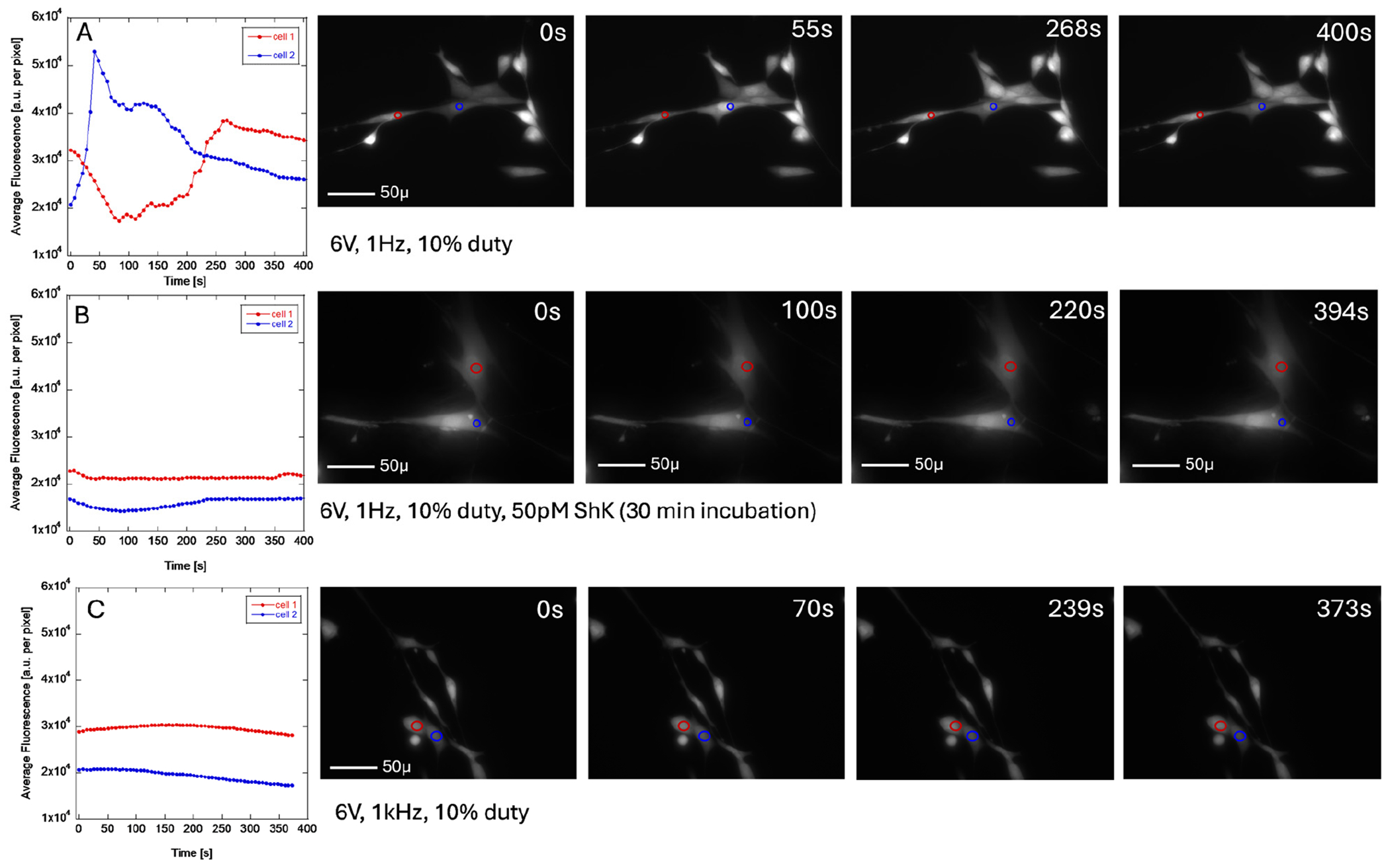
The time courses of fluorescence emission intensity in Human Dermal Fibroblast cells loaded passively with Fluo-4 averaged over the selected area (red or blue circle) in the image plotted next to the selected time frames; (**A**) treated with 6 V/1 Hz/10% duty ES, (**B**) treated with 50 pM ShK peptide 6 V/1 Hz/10% duty ES, (**C**) treated with 6 V/1 kHz/10% duty ES. The images and time courses were NOT corrected for background, initial point fluorescence emission, and artificial offsets induced by the solutions added to the culture during recording. The AVI files are available as Supplementary Materials (Video S2 A–C).

To observe the changes in intracellular calcium level concentrations in Human Dermal Fibroblasts during ES, the cells were incubated with Fluo-4 for 1 h before imaging and placed in live cell imaging solution with the GC electrodes attached to the top lid and lowered to the bottom of the dish. We used either house-made devices or IonOptix custom-made devices. Before the experiments were conducted, we measured the current passing through the cell to ensure that the current did not exceed 1 mA, which could damage the cells. The collected time-lapse images were analyzed in the same way as the control experiments. Figure 8 shows the changes in fluorescence intensity with time in two different cells (the area used for intensity calculations is indicated with the red or blue circle) treated with ES. Figure 8A shows the cells “firing” at different times during the stimulation with 1 Hz ES with 6 V positive bias (E field = 6 V/cm) and 10% duty. For clarity, the plot shows only two cells, but all cells show the same behavior (see time frames in Figure 8A). When the cells are preincubated with Shk peptide (50 pM, 30 min), this behavior is suppressed (Figure 8B), which indicates that the changes in intracellular calcium concentration are linked to the performance of Kv1.3 channel and are due to inward calcium current, rather than calcium release from the intracellular storage. When the Human Dermal Fibroblasts are stimulated using the same conditions but with 1 kHz frequency (Figure 8C), the fluorescence intensity stays almost constant, indicating small changes in the intracellular calcium concentration with time. While the changes are small, they are not as “flat” as the fluorescence intensity without the ES. This is an unexpected result since the ES stimulation with 1 Hz and 1 kHz frequencies results in a similar level of collagen expression. All video files are available in the Supplementary Materials section.

## 4. Discussion

The effects of prolonged electrical stimulation (ES) on Human Dermal Fibroblasts, such as cell migration, proliferation, and extracellular matrix (ECM) expression in vitro, have been established [4,14]. However, the tested experimental conditions are not easily transferable from the laboratory to clinical applications, particularly in the cosmetic industry. In this study, we address two major issues associated with the use of ES in Human Dermal Fibroblasts: the duration and frequency of ES, as well as the underlying mechanism of ES.

For comparison, we used two different frequencies of square wave pulses with a 10% duty cycle and a positive bias: 1 Hz and 1 kHz. Previous studies have shown that 1 Hz stimulation effectively upregulates collagen and elastin expression in vitro, provided the stimulation lasts for at least one hour [34]. Thus, a two-hour stimulation period allowed us to compare the effects of lower frequencies used often in laboratory settings with higher frequencies commonly used in clinical settings, for example, in Microcurrent Therapy. While Microcurrent Therapy is primarily used to stimulate muscles (located directly beneath the skin) to enhance ATP synthesis, it may also influence protein expression [43]. Indeed, we observed an upregulation of procollagen expression at similar levels (approximately 75%) in Human Dermal Fibroblast cells stimulated with either 1 Hz or 1 kHz pulses (Figure 2A) in vitro.

Another critical parameter is the duration of ES applied to Human Dermal Fibroblasts. In a laboratory setting, longer stimulation times improve sensitivity in detecting protein expression. However, extended stimulation is often impractical in clinical applications. To address this, we tested a 10-min ES duration with an added rest period between the termination of ES and cell lysis (first step in collagen assay) to allow for procollagen synthesis. We detected an increased collagen concentration after a 60-min rest period with both 1 Hz and 1 kHz frequencies. However, this increase (approximately 25%) was significantly lower than that observed after 2 h of ES. When the rest period was extended to 2 h, procollagen levels returned to those of non-stimulated cells. These findings align with our previous studies on Human Dermal Fibroblast cell morphology, where nuclear elongation caused by ES reverted after a two-hour rest period, bringing the cell back to homeostasis [34]. We conclude that higher-frequency ES in short-duration stimulation can enhance collagen expression, particularly when considered for potential daily treatments.

**Scheme 1.**
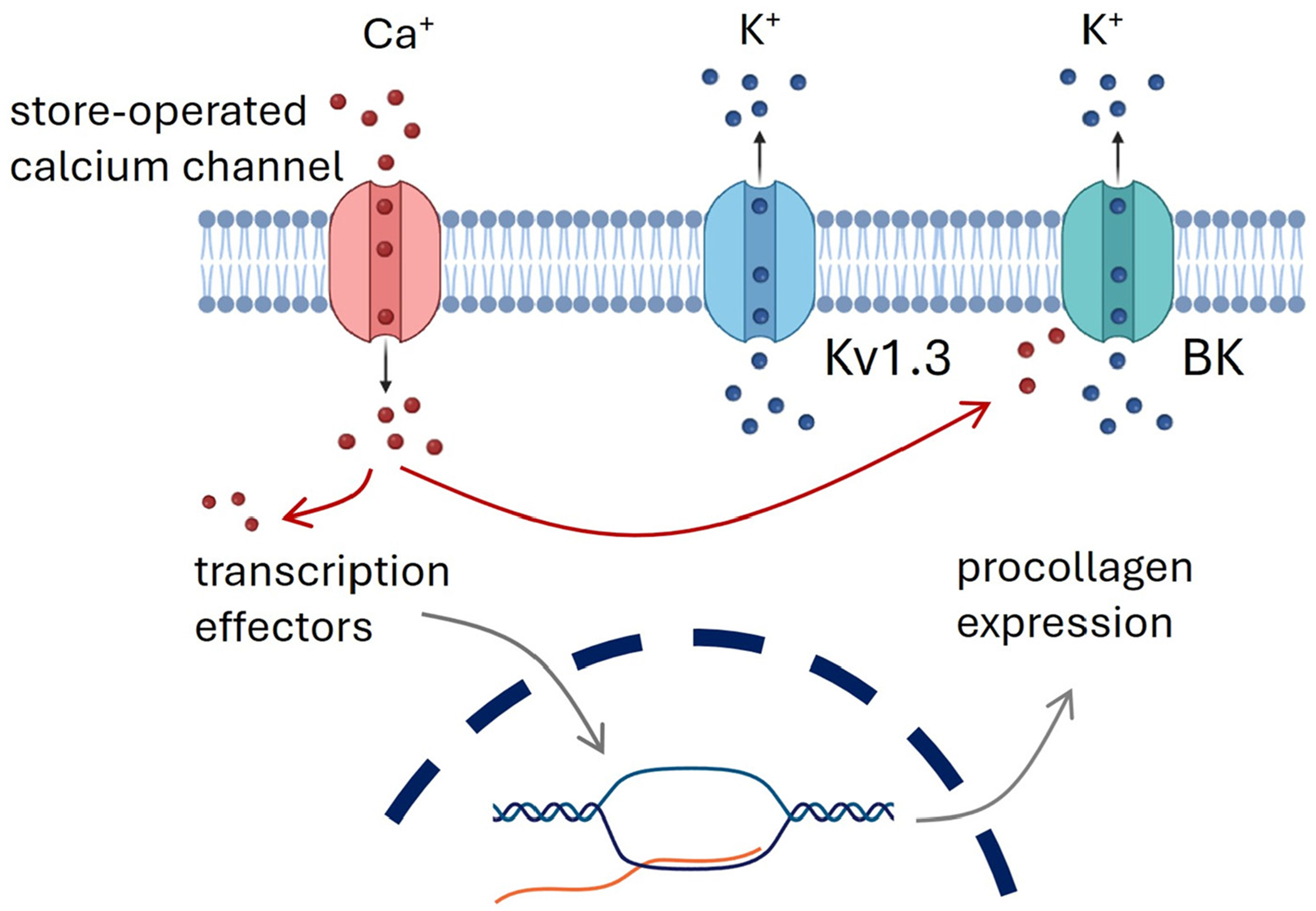
Proposed ES signal transduction pathway of Human Dermal Fibroblasts resulting in upregulation of collagen expression. BK = calcium-activated potassium channels.

The mechanism of ES in non-excitable cells remains poorly understood. This study aimed to elucidate the initial steps in signal transduction that lead to the transcriptional regulation of collagen synthesis and possibly other Extra Cellular Matrix proteins. Our primary hypothesis is that the electric field induces cell membrane polarization, which activates voltage-gated potassium channels, leading to calcium influx. The calcium influx causes the redistribution of intracellular free calcium ions that act as a secondary messenger to regulate transcriptional control (Scheme 1).

Applying ES to the Human Dermal Fibroblasts culture at 5 V/cm generates a potential difference of approximately 25–75 mV across the cell body (assuming a typical cell diameter of 50–150 μm of Human Dermal Fibroblasts in culture), which is sufficient to activate voltage-gated potassium channels. Using a set of ion channel blockers, 4-aminopyridine, Psoralen, and ShK peptide, we identified the Kv1.3 channel as a key sensor of ES. The application of Kv1.3 blockers resulted in decreased collagen expression (Figure 3), reduced cell proliferation (Figure 6), and visible morphological changes in the cells (Figure 5), all consistent with the disrupted function of Kv1.3. To confirm the identity of the channel, we used a fluorescently labeled 5FAM-ShK peptide toxin, which selectively binds Kv1.3 (Kd = 11 pM), demonstrating that Kv1.3 is the active channel in Human Dermal Fibroblasts. In future studies, we plan to validate this claim by using Western blot and RT-qPCR.

Previous studies on ion channel identification in Human Dermal Fibroblasts have detected calcium-activated potassium channels in all Human Dermal Fibroblast cells [25]. Thus, we assume calcium-activated potassium channels are present in Human Dermal Fibroblasts in our system as well. To support this claim, we increased calcium concentration in the media alongside the Kv1.3 blocker, which negated the blocker’s effects (Figure 6). This suggests that the increased extracellular calcium concentration raised the chemical potential (and an influx of calcium ions), activating the calcium-activated potassium channel. Additionally, we observed changes in intracellular calcium concentration following a spike addition of 1 mM calcium (Figure 7C), further supporting the idea that calcium-activated potassium channel activation is part of the ES effector pathway in Human Dermal Fibroblasts. However, we do not believe that calcium-activated potassium channels are directly activated by ES under our experimental conditions due to the low voltage amplitude applied in our experiments.

Store-operated calcium channels are present in all non-excitable cells and are thought to play a crucial role in cell–cell communication [44]. While ORAI1 is the most likely protein involved, identifying the specific store-operated calcium channel type is beyond the scope of this study. Thus, we only proceeded with the assumption that the cell surface of Human Dermal Fibroblasts has store-operated calcium channels present. Based on our observations, applying 1 Hz ES induces calcium influx and distinct changes in intracellular calcium concentration (Figure 8A). These changes were detected through differences in fluorescence intensity using Fluo-4 dye, which measures free intracellular calcium concentration. The observed calcium fluctuations occurred asynchronously between multiple cells during ES stimulation, unlike ionomycin treatment (Figure 7B), where all cells rapidly took up calcium simultaneously. Furthermore, when Human Dermal Fibroblast cells were preincubated with 50 pM ShK peptide (without the 5FAM tag) to block Kv1.3, no changes in intracellular calcium concentration were observed upon ES exposure (Figure 8B). This suggests that intracellular calcium fluctuations are controlled by the interplay between Kv1.3, calcium-activated potassium channels, and store-operated calcium channels. A similar Kv1.3/calcium-activated potassium channels/store-operated calcium channels pathway has been proposed by Teisseyre et al. as part of the “membrane potential model” regulating cell proliferation and protein expression in T lymphocytes [27]. Here, we confirmed the relationship between ES, Kv1.3, and intracellular calcium concentration, which may contribute to transcriptional control. However, we did not identify the specific pathway through which calcium acts as a secondary messenger.

Interestingly, when Human Dermal Fibroblast cells were exposed to ES under the same experimental conditions but with an increased field frequency (1 kHz instead of 1 Hz), the changes in intracellular calcium concentration were very small (Figure 8C). Calcium levels fluctuated slightly over time compared to control experiments without stimulation (Figure 7A). Despite this observation, collagen expression levels were elevated (Figure 2) under both 1 Hz and 1 kHz stimulation, with no significant differences between the two frequencies. We identify two possible explanations for this behavior.

First, our experiment may not have captured potential intracellular calcium changes induced by 1 kHz ES, as “calcium waves” might be too rapid for our imaging acquisition rate, or the Fluo-4 dye may not respond quickly enough to detect field-induced fluctuations [45].

Second, we cannot rule out the possibility that high-frequency ES follows a different stimulation mechanism than low-frequency (1 Hz) ES. Sun et al. [46] calculated that calcium influx rates decrease significantly below 100 Hz ES based on experimental data from the Cho group [47]. While the calcium influx may not be very effective in high-frequency ES, the effectiveness of stimulation at higher frequencies may be attributed to lower membrane impedance, decreased by two orders of magnitude using either 1 Hz or 1 kHz rather than Kv1.3 activation [18]. In the future, we plan to investigate the mechanism of high-frequency ES of Human Dermal Fibroblasts, given that high-frequency ES is effective in several currently used cosmetic applications, such as high-frequency or radio-frequency therapy.

## 5. Conclusions

This study demonstrates that both low-frequency (1 Hz) and high-frequency (1 kHz) electrical stimulation (ES) can enhance procollagen expression in Human Dermal Fibroblasts, although the underlying mechanisms may differ. Short-duration ES (10 min) followed by a rest period was sufficient to moderately increase collagen synthesis, though extended stimulation (120 min) shows significantly greater effects. While 1 Hz ES appears to function through a calcium-dependent pathway involving the activation of Kv1.3 channels, the mechanism behind 1 kHz ES remains less clear, potentially involving alternative pathways. These findings suggest that high-frequency, short-duration ES protocols may be viable for cosmetic applications due to their practicality and efficacy, and further research is warranted to elucidate the distinct molecular mechanisms behind high-frequency ES in non-excitable cells.

## Supplementary Material

Supplementary Figures

Supplementary Videos

## Data Availability

The data presented in this study are available upon request from the corresponding author, including software used for time laps fluorescence analysis.

## Abbreviations

The following abbreviations are used in this manuscript:

Kv: Voltage-Gated Potassium Channels
ES: Electrical Stimulation
IC50: Half Maximal Inhibitory Concentration
ShK: Sea Anemone Toxin (Kv1.3 Channel Blocker)
5FAM: 5-Carboxy Fluorescein
Kv1.3: Voltage-Gated Potassium Channel 1.3
Fluo-4: Fluorescent Dye For Calcium Imaging
ORAI1: Calcium Release-Activated Calcium Channel Protein 1
