# Supplementary material for: Kv1.3 Ion Channels Mediate Electrical Stimulation-Induced Collagen Expression in Human Dermal Fibroblasts
Source: Cosmetics. Author manuscript; Available in PMC 2025 Aug 29. (PMC12393046; doi:10.3390/cosmetics12030086)
Supplement: Supplementary Figures [file NIHMS2079742-supplement-Supplementary_Figures.pdf]

Supplementary Materials

Kv1.3 Ion Channels Participate in Transduction of Electrical Stimulation in Human Dermal Fibroblasts Resulting in Upregulation of Collagen Expression

Catherine Obiajulu[1], Diem Nguyen[1], Kim Bui[1], Timothy Tran[1], Annamarie Vu[1], Cortney Ngo[1], Ian A. Slowinski[2], Kazuyuki Miyazawa[3], Katarzyna Slowinska[1]*

[1] Department of Chemistry and Biochemistry, California State University Long Beach, 1250 Bellflower Blvd, Long Beach, California 90840
[2] Department of Mechanical Engineering, Purdue University, 585 Purdue Mall, West Lafayette, Indiana 47907
[3] Mirai Technology Institute, Shiseido Co. LTD, 1-2-11, Takashima, Nishi-ku, Yokohama, Japan

Content:

1. Supplementary Figures

Figure S1: Impedance Measurement for ES Reactor
Figure S2: Calibration of Collagen Concentration (Sircol Assay)
Figure S3: HDF cell morphology before and after ES

2. Supplementary Videos

Video S1: Time laps videos for Figure 7
The time courses of Fluorescence emission intensity in HDFa cells loaded passively with Fluo-4 (FITC channel); (S1A) baseline, (S1B) addition of 1 μM ionomycin at 167s, (S1C) addition of 1 mM calcium at 65s.

Video S2: Time laps videos for Figure 8
The time courses of fluorescence emission intensity in HDFa cells loaded passively with Fluo-4 (FITC channel); (S2A) treated with 6 V/1 Hz/10% duty ES, (S2B) treated with 50pM ShK peptide 6V/1Hz/10% duty ES, (S2C) treated with 6 V/1 kHz/10% duty ES.

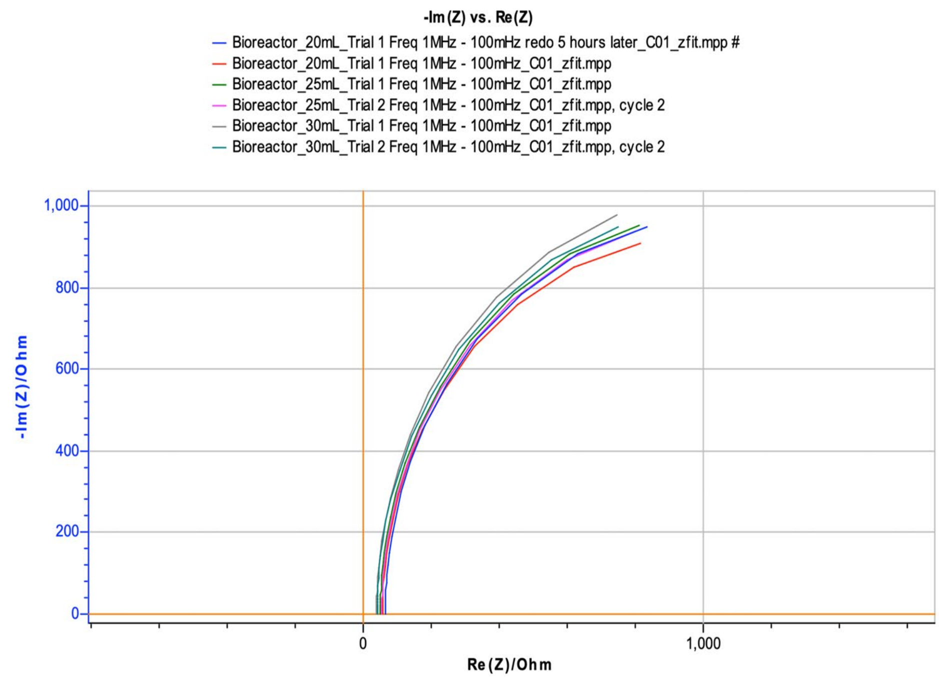

Figure S1. Impedance Measurement for ES Reactor.

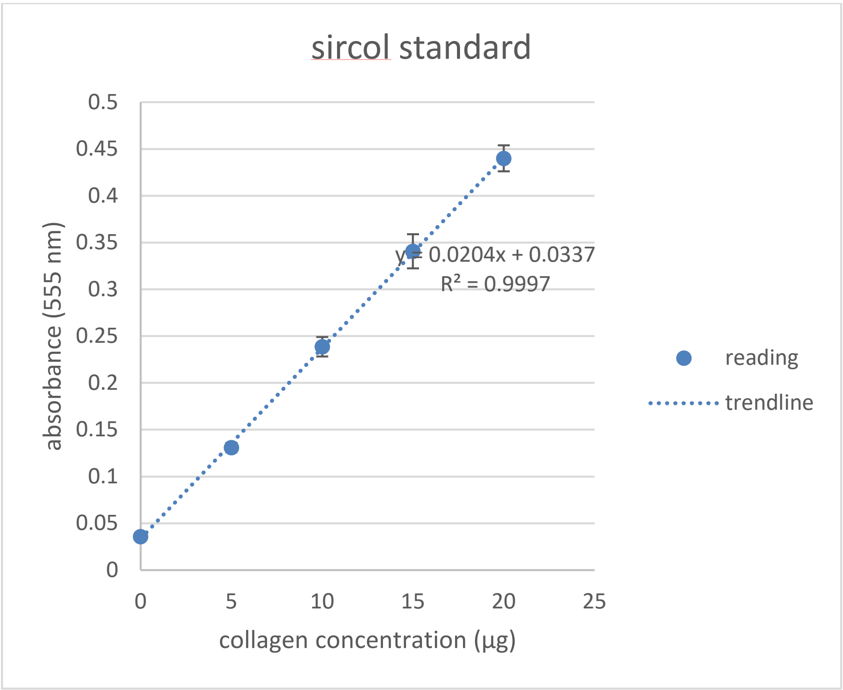

Figure S2. Calibration of Collagen Concentration (Sircol Assay).

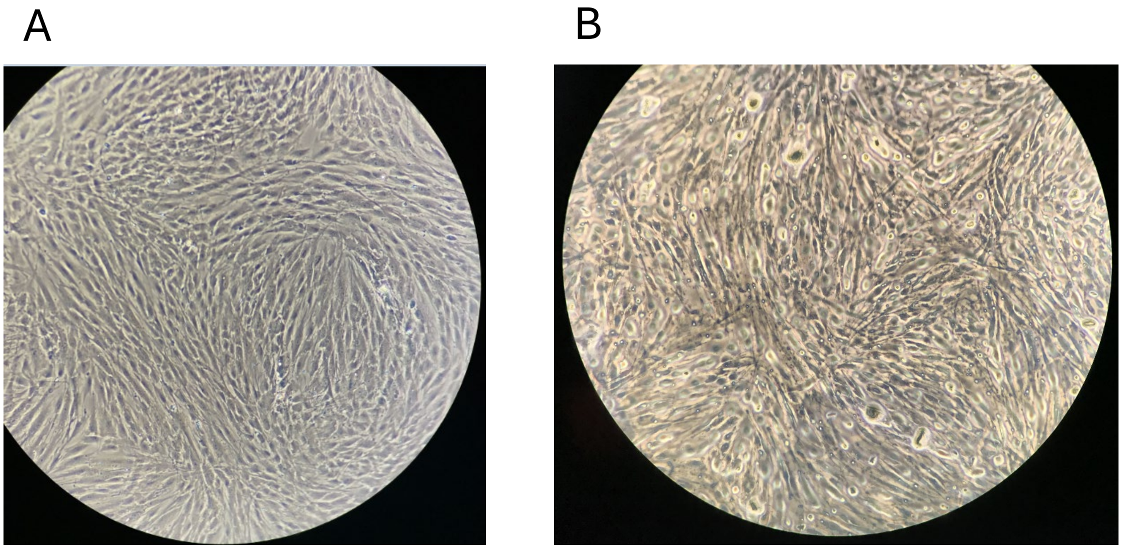

Figure S3. Bright field images of HDFa (x10) before (A) and after (B) ES: 4h, positive bias +5V, 1% duty, 1Hz) incubated with trypan blue.
